# Monitoring sodium content in packaged foods sold in the Americas and compliance with the updated regional sodium reduction targets

**DOI:** 10.1371/journal.pone.0304922

**Published:** 2025-04-03

**Authors:** Yahan Yang, Nadia Flexner, Maria Victoria Tiscornia, Leila Guarnieri, Adriana Blanco-Metzler, Hilda Núñez-Rivas, Marlene Roselló-Araya, Paola Arévalo-Rodríguez, Maria Fernanda Kroker-Lobos, Francisco Diez-Canseco, Mayra Meza-Hernández, Kiomi Yabiku-Soto, Lorena Saavedra-Garcia, Lorena Allemandi, Leo Nederveen, Mary R. L’Abbé

**Affiliations:** 1 Department of Nutritional Sciences, Temerty Faculty of Medicine, University of Toronto, Toronto, Canada; 2 Global Health Advocacy Incubator, Washington, District of Columbia, United States of America; 3 Fundación Interamericana del Corazón Argentina, Buenos Aires, Argentina; 4 Costa Rican Institute of Research and Education on Nutrition and Health (INCIENSA), Tres Ríos, Cartago, Costa Rica; 5 INCAP Research Center for the Prevention of Chronic Diseases, Institute of Nutrition of Central America and Panama, Guatemala City, Guatemala, United States of America; 6 CRONICAS Center of Excellence in Chronic Diseases, Universidad Peruana Cayetano Heredia, Lima, Peru,; 7 Pan American Health Organization/World Health Organization, Washington, District of Columbia, United States of America; Center for Research and Technology Transfer, VIETNAM

## Abstract

**Background:**

Sodium reduction is a cost-effective measure to prevent noncommunicable diseases. The World Health Organization (WHO) established a target of a 30% relative reduction in mean population intake of sodium by 2025. The Pan American Health Organization (PAHO) published sodium reduction targets (SRTs) for packaged foods in 2015, expanding and updating the targets in 2021 to help Member States with its efforts in reducing population sodium intake.

**Objective:**

This study examined the current sodium levels in packaged foods among five countries in the Americas and monitored cross-sectional and longitudinal compliance with the sodium targets from 2015 to 2022.

**Methods:**

Food labels were systematically collected from the main supermarkets in five countries in the Americas region in 2022. Sodium levels per 100g and per kcal for collected food labels in 16 PAHO categories and 75 subcategories were analyzed and compared against the updated SRTs. Further analysis of three countries that have longitudinal data for 2015–2016, 2017–2018 and 2022 was conducted to compare sodium per 100 g against the 2015 SRTs.

**Results:**

A total of 25,569 food items were analyzed. Overall, *‘processed meat and poultry’* had the highest sodium levels, although there were large variations within categories. 47% and 45% of products met the sodium per 100g and per kcal 2022 SRTs, respectively. Peru had the highest compliance, whereas Panama had the lowest for both targets. Among Argentina, Costa Rica and Peru, the proportion of foods meeting the 2015 PAHO lower targets were 48, 53 and 61% for 2015–2016, 2017–2018 and 2022, respectively (p < 0.001).

**Conclusions:**

This study showed that around half of the examined foods met their respective SRTs and there have been small improvements in compliance over time. Further efforts are required to reach the WHO’s global sodium reduction goal by 2025, such as implementation of mandatory SRTs and front-of-pack labelling regulations.

## Introduction

Hypertension is one of the major risk factors for cardiovascular diseases (CVDs) and it has been estimated to account for 10.8 million deaths globally in 2019 [1]^,^[2]. The number of adults with hypertension has been increasing in the past few decades, with more than 1 billion adults affected by hypertension in 2019, globally [3]. It has been well demonstrated that excessive sodium intake is a significant causal risk factor for the development of hypertension, and reducing dietary sodium intake can have a favorable effect on the cardiovascular system [4]. The World Health Organization (WHO) published a guideline in 2012 recommending less than 5g of salt (2g of sodium) intake per day for adults [5]. However, the global average salt intake in 2019 was estimated to be more than double the recommendation at 10.8g of salt (4.3g of sodium) per day [1].

In 2013, WHO established an action plan that included reducing salt intake by 30% by 2025, and all 194 Member States agreed to it [6]. A series of interventions has been proposed, including reformulating foods to contain less sodium, establishing public food procurement policies to limit high-sodium foods, implementing mandatory front-of-package labelling to inform consumers about products high in sodium (along with other nutrients of concern), and using mass media campaigns to reduce sodium intake [7]. However, a report released in early 2023 showed that the world is off-track to achieve this global target, with only 5% of countries (Brazil, Chile, Czech Republic, Lithuania, Malaysia, Mexico, Saudi Arabia, Spain and Uruguay) implementing at least two mandatory and comprehensive sodium reduction policies and all WHO sodium-related ‘best buys’[1].

To support the Americas region in accomplishing the global target, the Pan American Health Organization (PAHO) published a set of regional SRTs (2015 PAHO targets) in 2015 for 18 food categories that were commonly sold in the region [8]. In 2019, a study examined the sodium levels in packaged foods sold in 14 Latin American and Caribbean countries (LAC) according to the 2015 PAHO targets. The study found 82% compliance with the regional target and 47% compliance with the lower and stricter targets [9]. The regional target level was the maximum level (mg/100g) or upper limit, whereas food manufacturers were encouraged to reach the lower target level that is reflective of the average level in reference countries [8]. A later study published in 2021 showed that compliance with regional targets increased from 83% to 89% from 2015–2016 to 2017–2018 among LAC [10]. Therefore, based on sodium levels in packaged foods in the region, PAHO updated the targets in 2021 to establish progressive regional SRTs for 2022 and 2025 (the 2022 and 2025 PAHO targets). Additionally, it categorized foods into 16 main categories and 75 subcategories [11,12]. Similarly, WHO published global sodium benchmarks in 2021 and subsequently updated them in 2024, defined as the lowest maximum values for each subcategory based on existing national or regional targets, for sodium levels across different food categories [13,14]. However, there has been no further follow-up study monitoring sodium levels in packaged foods in the Americas since 2018. Thus, the objectives of this study were to 1) examine the current sodium levels among five countries in the Americas (Argentina, Canada, Costa Rica, Panama, and Peru), and monitor compliance with the Updated PAHO Regional SRTs for 2022; and 2) monitor the progress of sodium reduction among three LAC (Argentina, Costa Rica and Peru) that have longitudinal data.

## Methods

This cross-sectional study was conducted using data from five countries: Argentina, Canada, Costa Rica, Panama, and Peru. Data from the Nutrition Facts table (NFt) (n = 44,570) were collected in supermarkets in Argentina (n = 4,340), Costa Rica (7,402), Panama (n = 1,509) and Peru (n = 5,378) between March and August 2022. Foods were selected from one or more of the major supermarket chains from different socioeconomic status in each country, representing a comprehensive sample of the packaged foods across different socioeconomic groups. The Food Label Information Program for Latin America and Caribbean countries (FLIP-LAC) was used for data collection and analyses. FLIP-LAC is a smartphone-based technology and web database, and the methodology was developed by The University of Toronto (U of T) [15]. The Canadian data (n = 25,941) were extracted from the Food Label Information and Price (FLIP) database collected in 2020 [15].

Foods were classified into 16 major categories and 75 subcategories described in the Updated PAHO Regional SRTs [12]. The updated PAHO target food categorization was individually completed by each country team and the U of T research team validated the results and resolved discrepancies with country team members. The sodium content was standardized into mg/100g and mg/kcal where data were available. Foods excluded from the analyses included duplicated items, foods that could not be categorized under the PAHO categories (n = 19,001), and foods that have 0 kcal were additionally excluded from the mg/kcal analysis, due to mathematical restrictions (n = 387). Summary statistics were calculated for food products by food categories and by country. The sodium level was compared against the Updated PAHO Regional SRTs (Supporting information S1 Table) to determine the proportion of foods that met or exceeded the target level set for 2022.

Since three countries in this study had baseline data from 2015–2016 and 2017–2018, categorized under the 2015 PAHO targets, a further sub-analysis was conducted by categorizing the foods collected in 2022 from Argentina, Costa Rica and Peru into the 2015 PAHO target categories (18 food categories) [8]. Sodium levels were compared against the 2015 PAHO regional (upper) target level as well as the lower target level. Proportion of foods meeting the regional targets was compared among the three timepoints. Comparisons between the 2015–2016 and 2022 were analyzed using a Chi-Square test, or Fisher’s exact test for cells with < 5 counts. All analyses were conducted with R studio (5.12.10) and Microsoft Excel (2016).

## Results

This study included a total of 25,569 items across 16 PAHO sodium reduction main categories and 75 subcategories (Argentina n = 2,515, Canada n = 15,268, Costa Rica n = 3,875, Panama n = 1,121, and Peru n = 2,790).

### Sodium levels per 100g and per kcal by PAHO categories

For the per 100g analysis, 23,663 foods were assessed. *‘Processed meat and poultry’* and *‘sauces, dips, gravy, and condiments’* had the highest median sodium levels per 100g (both at 800mg/100g), followed by *‘fats and oils’* (720mg/100g) (Table 1). The variations within categories were high, particularly among *‘sauces, dips, gravy and condiments’* (SD =  5,733mg/100g), *‘processed fish and seafood’* (SD =  972mg/100g) and *‘processed vegetables, beans and legumes’* (SD =  599mg/100g). There were variations between the median sodium levels between countries, with the largest variation within *‘sauces, dips, gravy, and condiments’*, *‘soy products and meat alternatives’* and *‘processed meat and poultry’*. The lowest variation between countries was within *‘fresh or dried plain pasta and noodles’*, *‘processed fish and seafood’* and *‘ready-made foods’*.

**Table 1:** Distribution of sodium content (mg) per 100g/ml of packaged foods per PAHO food category at the regional level and by country.

Category	Country	Products (n)	Mean (mg)	SD (mg)	Minimum (mg)	q25 (mg)	Median (mg)	q75 (mg)	Maximum (mg)
1. Bread, bread products and crisp breads	**Regional**	**1564**	**504**	**309**	**0**	**364**	**467**	**600**	**5300**
	Argentina	226	476	288	0	365	457	608	2902
	Canada	1019	514	233	0	375	483	600	1700
	Costa Rica	188	551	615	0	291	473	658	5300
	Panama	44	527	154	240	444	500	592	1040
	Peru	87	354	113	40	311	365	396	676
2. Cakes, biscuits, pastries, and sweet breads	**Regional**	**3530**	**371**	**269**	**0**	**191**	**308**	**469**	**2600**
	Argentina	626	316	254	0	129	251	430	2110
	Canada	2028	400	280	0	224	326	500	2600
	Costa Rica	512	348	248	0	179	280	442	1214
	Panama	99	415	313	34	201	354	537	1667
	Peru	265	303	190	0	179	267	367	1063
3. Corn derivatives	**Regional**	**58**	**476**	**447**	**0**	**103**	**349**	**834**	**1600**
	Costa Rica	43	513	487	0	75	396	847	1600
	Panama	8	348	336	15	115	272	479	983
	Peru	7	399	270	63	343	344	368	960
4. Breakfast cereal	**Regional**	**1089**	**294**	**223**	**0**	**98**	**283**	**442**	**2388**
	Argentina	87	232	177	0	77	240	323	810
	Canada	617	325	234	0	105	364	500	2388
	Costa Rica	195	278	206	0	113	268	374	1307
	Panama	48	339	215	0	205	350	408	1133
	Peru	142	202	187	0	18	183	347	775
5. Savoury snacks	**Regional**	**1692**	**545**	**354**	**0**	**305**	**500**	**731**	**3000**
	Argentina	143	602	250	0	429	604	780	1292
	Canada	962	556	348	0	326	520	724	3000
	Costa Rica	336	516	390	0	202	445	720	2467
	Panama	92	646	467	27	358	532	842	2491
	Peru	159	434	276	19	266	359	577	1434
6. Cheese	**Regional**	**1813**	**696**	**421**	**0**	**475**	**667**	**800**	**7143**
	Argentina	256	642	425	0	346	577	877	3300
	Canada	1167	698	385	0	500	667	767	3400
	Costa Rica	214	717	584	0	506	641	889	7143
	Panama	68	898	423	32	634	780	1253	1800
	Peru	108	629	348	10	398	608	800	1800
7. Processed vegetables, beans, and legumes	**Regional**	**1976**	**546**	**599**	**0**	**168**	**326**	**767**	**4400**
	Argentina	151	677	875	0	73	240	1148	2885
	Canada	1244	539	582	0	153	324	767	4333
	Costa Rica	394	519	508	0	220	346	640	3500
	Panama	109	521	657	0	232	280	460	4400
	Peru	78	577	536	4	200	360	929	2300
8. Processed meat and poultry	**Regional**	**1776**	**896**	**567**	**0**	**527**	**800**	**1036**	**5460**
	Argentina	178	962	747	46	593	753	1036	5460
	Canada	1210	881	506	0	511	800	1026	3143
	Costa Rica	184	1071	759	5	722	908	1225	4800
	Panama	91	790	394	96	626	769	969	2495
	Peru	113	763	546	198	335	600	900	2640
9. Processed fish and seafood	**Regional**	**946**	**514**	**972**	**0**	**255**	**354**	**487**	**9940**
	Argentina	54	541	1085	0	205	303	457	5913
	Canada	579	554	1132	0	255	368	504	9940
	Costa Rica	175	477	681	22	275	345	491	5938
	Panama	72	366	178	10	271	360	446	985
	Peru	66	403	227	70	269	350	450	1152
10. Soy products and meat alternatives	**Regional**	**274**	**478**	**278**	**0**	**355**	**421**	**577**	**1929**
	Argentina	77	382	120	0	328	376	433	784
	Canada	116	555	344	0	393	473	632	1929
	Costa Rica	50	547	236	5	400	539	678	1375
	Panama	5	204	276	6	12	24	360	620
	Peru	26	339	177	15	301	360	386	680
11. Soups	**Regional**	**715**	**316**	**302**	**0**	**216**	**276**	**336**	**4538**
	Argentina	45	319	138	32	259	285	341	702
	Canada	485	264	125	0	201	260	320	792
	Costa Rica	83	356	358	18	221	309	351	3250
	Panama	48	303	282	62	188	287	323	1797
	Peru	54	727	776	97	328	405	953	4538
12. Ready-made foods, convenience foods, and mixed dishes	**Regional**	**2133**	**412**	**283**	**0**	**254**	**341**	**486**	**3000**
	Argentina	144	412	174	31	286	397	519	973
	Canada	1784	400	260	0	250	333	464	2050
	Costa Rica	116	493	398	0	160	421	726	1900
	Panama	24	497	322	0	309	440	749	1200
	Peru	65	570	589	85	317	380	530	3000
13. Fresh or dried plain pasta and noodles	**Regional**	**1108**	**20**	**101**	**0**	**0**	**3**	**11**	**2040**
	Argentina	202	16	35	0	9	10	11	216
	Canada	571	22	99	0	0	0	12	1789
	Costa Rica	125	32	195	0	0	0	2	2040
	Panama	39	7	10	0	0	4	11	36
	Peru	171	12	62	0	0	2	5	770
14. Granola and energy bars and nut butters/spreads	**Regional**	**967**	**222**	**146**	**0**	**115**	**221**	**313**	**893**
	Argentina	33	137	119	0	58	104	208	429
	Canada	736	235	140	0	136	232	320	867
	Costa Rica	117	188	170	0	60	182	280	893
	Panama	31	285	138	56	156	281	391	500
	Peru	50	138	122	0	34	83	280	391
15. Fats and oils	**Regional**	**1044**	**718**	**435**	**0**	**567**	**720**	**867**	**11000**
	Argentina	86	542	472	0	110	629	806	2520
	Canada	692	730	243	0	600	733	867	1800
	Costa Rica	144	736	929	0	415	673	900	11000
	Panama	48	835	235	383	692	817	972	1867
	Peru	74	708	334	0	400	700	884	1571
16. Sauces, dips, gravy and condiments	**Regional**	**2978**	**3157**	**5733**	**0**	**400**	**800**	**2800**	**36000**
	Argentina	203	1946	3974	0	275	483	1283	24000
	Canada	2058	3149	5493	0	417	833	3200	36000
	Costa Rica	341	3222	6367	0	385	824	2333	32750
	Panama	153	4533	7137	0	528	1110	4550	34667
	Peru	223	3290	6874	0	362	681	1413	32660
Total	**Regional**	23663	820	2261	0	233	402	714	36000
	Argentina	2511	570	1286	0	152	360	640	24000
	Canada	15268	853	2251	0	256	429	733	36000
	Costa Rica	3217	782	2292	0	200	393	721	32750
	Panama	979	1153	3194	0	269	460	829	34667
	Peru	1688	781	2705	0	172	350	610	32660

For the per kcal analysis, 23,236 foods were assessed. *‘Sauces, dips, gravy, and condiments’*, *‘soups’* and *‘processed vegetables, beans, and legumes’* have the highest median sodium level per kcal of 7.8mg/kcal, 6.0mg/kcal and 5.1mg/kcal, respectively (Table 2). The lowest median sodium levels per kcal were among *‘fresh or dried plain pasta and noodles’* (0mg/kcal), *‘granola and energy bars and nut butters/spreads’* (0.5mg/kcal) and *‘cakes, biscuits, pastries, and sweet breads’* (0.7mg/kcal). The largest variations between countries were found among *‘sauces, dips gravy and condiments’*, *‘soups’* and *‘soy products and meat alternatives*. Variation within category was highest among *‘sauces, dips, gravy, and condiments’* (SD = 41.4mg/kcal), *‘processed vegetables, beans and legumes’* (SD = 20.2mg/kcal) and *‘soups’* (SD = 17.8mg/kcal).

**Table 2:** Distribution of sodium content (mg) per kcal of packaged foods, by PAHO category at the regional level and by country.

Category	Country	Product (n)	Mean (mg)	SD (mg)	Minimum (mg)	q25 (mg)	Median (mg)	q75 (mg)	Maximum (mg)
1. Bread, bread products and crisp breads	**Regional**	**1556**	**1.7**	**1.0**	**0.0**	**1.2**	**1.6**	**2.0**	**17.7**
	Argentina	226	1.6	0.8	0.0	1.3	1.7	2.0	5.0
	Canada	1011	1.7	0.7	0.0	1.3	1.6	2.0	5.0
	Costa Rica	187	1.8	2.0	0.0	0.9	1.6	2.2	17.7
	Panama	45	1.8	0.7	0.8	1.5	1.7	2.1	4.7
	Peru	87	1.2	0.5	0.1	0.9	1.3	1.5	2.4
2. Cakes, biscuits, pastries, and sweet breads	**Regional**	**3524**	**0.9**	**0.8**	**0.0**	**0.5**	**0.7**	**1.3**	**23.0**
	Argentina	625	0.8	0.6	0.0	0.3	0.6	1.1	4.6
	Canada	2024	1.0	0.9	0.0	0.5	0.8	1.4	23.0
	Costa Rica	511	0.9	0.7	0.0	0.4	0.7	1.2	3.5
	Panama	99	1.0	0.9	0.1	0.5	0.8	1.3	5.0
	Peru	265	0.7	0.4	0.0	0.4	0.6	0.9	2.3
3. Corn derivatives	**Regional**	**58**	**1.3**	**1.2**	**0.0**	**0.5**	**1.0**	**2.0**	**6.3**
	Costa Rica	43	1.2	1.0	0.0	0.2	0.8	2.0	3.2
	Panama	8	2.0	2.1	0.1	0.5	1.3	2.7	6.3
	Peru	7	1.1	0.4	0.9	0.9	1.0	1.0	2.1
4. Breakfast cereal	**Regional**	**1090**	**0.8**	**0.6**	**0.0**	**0.3**	**0.8**	**1.2**	**6.8**
	Argentina	87	0.6	0.5	0.0	0.2	0.6	0.9	2.2
	Canada	617	0.9	0.6	0.0	0.3	1.0	1.3	6.8
	Costa Rica	195	0.7	0.5	0.0	0.3	0.7	1.1	2.5
	Panama	49	0.9	0.6	0.0	0.5	0.9	1.1	3.1
	Peru	142	0.5	0.5	0.0	0.0	0.5	0.9	2.1
5. Savoury snacks	**Regional**	**1691**	**1.1**	**0.8**	**0.0**	**0.6**	**1.0**	**1.4**	**6.7**
	Argentina	143	1.2	0.6	0.0	0.8	1.2	1.7	2.9
	Canada	960	1.1	0.7	0.0	0.6	1.0	1.4	6.7
	Costa Rica	336	1.0	0.9	0.0	0.4	0.8	1.4	5.4
	Panama	93	1.2	0.9	0.0	0.7	1.0	1.6	4.4
	Peru	159	0.9	0.6	0.0	0.5	0.7	1.1	3.2
6. Cheese	**Regional**	**1807**	**2.3**	**1.5**	**0.0**	**1.5**	**1.9**	**2.6**	**25.0**
	Argentina	256	2.1	1.3	0.0	1.3	1.9	2.6	9.7
	Canada	1162	2.3	1.3	0.0	1.6	1.9	2.5	10.3
	Costa Rica	214	2.4	2.4	0.0	1.5	1.9	2.7	25.0
	Panama	68	3.0	1.7	0.2	1.7	2.4	4.1	7.5
	Peru	107	2.1	1.2	0.0	1.3	1.8	2.8	6.4
7. Processed vegetables, beans, and legumes	**Regional**	**1933**	**11.6**	**20.2**	**0.0**	**2.3**	**5.1**	**10.6**	**192.0**
	Argentina	148	9.4	15.0	0.0	1.3	3.9	12.4	123.1
	Canada	1221	12.7	22.1	0.0	2.2	4.9	10.7	192.0
	Costa Rica	376	8.6	11.8	0.0	3.2	5.3	9.3	114.3
	Panama	110	11.7	27.5	0.0	3.2	5.8	9.0	186.7
	Peru	78	12.1	14.4	0.1	3.0	6.3	17.4	84.6
8. Processed meat and poultry	**Regional**	**1772**	**4.3**	**2.8**	**0.0**	**2.4**	**3.6**	**5.6**	**23.7**
	Argentina	178	4.6	3.7	0.3	2.2	3.4	5.5	23.7
	Canada	1204	4.2	2.4	0.0	2.3	3.6	5.5	12.4
	Costa Rica	184	5.3	3.8	0.0	3.1	4.2	7.0	23.4
	Panama	93	4.5	2.6	0.4	2.9	4.2	5.8	12.6
	Peru	113	4.0	2.8	0.8	2.0	3.3	4.6	14.6
9. Processed fish and seafood	**Regional**	**945**	**3.5**	**7.1**	**0.0**	**1.4**	**2.2**	**3.5**	**76.5**
	Argentina	54	3.5	6.4	0.0	1.3	2.2	3.0	40.3
	Canada	579	4.0	8.6	0.0	1.5	2.4	3.6	76.5
	Costa Rica	175	2.7	3.5	0.1	1.2	1.9	3.1	31.7
	Panama	71	2.9	1.9	0.5	1.4	2.4	3.9	12.3
	Peru	66	2.5	1.8	0.2	1.4	2.0	3.2	9.1
10. Soy products and meat alternatives	**Regional**	**272**	**2.7**	**1.5**	**0.0**	**1.8**	**2.5**	**3.4**	**8.2**
	Argentina	77	2.2	1.1	0.0	1.3	2.1	2.5	6.5
	Canada	115	3.2	1.4	0.0	2.3	3.0	4.0	7.0
	Costa Rica	49	3.1	1.8	0.1	2.3	2.7	3.7	8.2
	Panama	5	1.4	2.3	0.1	0.1	0.1	1.3	5.5
	Peru	26	1.9	0.9	0.1	1.6	2.4	2.6	2.8
11. Soups	**Regional**	**700**	**11.7**	**17.8**	**0.0**	**3.9**	**6.0**	**11.1**	**180.0**
	Argentina	45	10.5	8.0	2.2	3.7	9.9	12.1	40.7
	Canada	472	12.5	20.4	0.0	3.8	6.0	8.6	180.0
	Costa Rica	83	12.9	13.6	0.0	4.9	8.7	14.8	66.2
	Panama	48	10.7	6.8	2.0	4.4	10.8	16.2	26.5
	Peru	52	5.5	3.7	0.9	3.2	4.5	6.2	17.6
12. Ready-made foods, convenience foods, and mixed dishes	**Regional**	**2116**	**2.3**	**1.2**	**0.0**	**1.6**	**2.1**	**2.7**	**21.8**
	Argentina	144	2.0	0.8	0.2	1.4	2.1	2.5	4.8
	Canada	1769	2.2	1.1	0.0	1.6	2.1	2.6	21.8
	Costa Rica	115	2.3	1.4	0.0	1.3	2.1	3.0	6.7
	Panama	24	2.6	3.2	0.0	2.0	2.2	2.5	16.9
	Peru	64	3.2	2.2	0.3	1.9	2.7	3.7	14.1
13. Fresh or dried plain pasta and noodles	**Regional**	**1110**	**0.1**	**0.3**	**0.0**	**0.0**	**0.0**	**0.0**	**5.1**
	Argentina	201	0.0	0.1	0.0	0.0	0.0	0.0	0.8
	Canada	571	0.1	0.3	0.0	0.0	0.0	0.0	5.1
	Costa Rica	128	0.1	0.6	0.0	0.0	0.0	0.0	5.1
	Panama	39	0.1	0.2	0.0	0.0	0.0	0.0	0.7
	Peru	171	0.0	0.2	0.0	0.0	0.0	0.0	2.1
14. Granola and energy bars and nut butters/spreads	**Regional**	**967**	**0.5**	**0.4**	**0.0**	**0.3**	**0.5**	**0.8**	**4.1**
	Argentina	33	0.3	0.3	0.0	0.1	0.3	0.5	0.9
	Canada	736	0.6	0.3	0.0	0.3	0.6	0.8	3.0
	Costa Rica	117	0.5	0.6	0.0	0.1	0.4	0.7	4.1
	Panama	32	0.6	0.2	0.1	0.4	0.6	0.8	0.9
	Peru	49	0.3	0.3	0.0	0.1	0.2	0.5	1.1
15. Fats and oils	**Regional**	**1035**	**2.6**	**3.9**	**0.0**	**1.0**	**1.8**	**3.1**	**44.0**
	Argentina	85	2.0	2.0	0.0	0.5	1.6	3.0	10.5
	Canada	691	2.6	3.7	0.0	1.0	1.8	3.1	36.0
	Costa Rica	140	3.4	5.7	0.0	1.1	2.0	3.3	44.0
	Panama	45	2.9	4.3	0.6	1.2	2.0	3.3	28.0
	Peru	74	2.7	3.3	0.0	1.0	1.6	2.7	22.7
16. Sauces, dips, gravy and condiments	**Regional**	**2660**	**22.0**	**41.4**	**0.0**	**3.7**	**7.8**	**19.0**	**653.2**
	Argentina	190	28.8	52.2	0.0	3.0	8.5	36.0	455.0
	Canada	1819	18.3	30.2	0.0	3.8	7.7	18.0	334.7
	Costa Rica	291	23.6	42.9	0.0	2.9	8.0	22.2	335.0
	Panama	153	45.9	70.1	0.0	5.5	14.2	50.0	352.5
	Peru	207	28.0	68.8	0.0	2.6	6.3	16.2	653.2
Total	**Regional**	**23236**	**5.2**	**17.0**	**0.0**	**0.8**	**1.7**	**3.5**	**653.2**
	Argentina	2492	4.3	16.7	0.0	0.5	1.4	2.5	455.0
	Canada	14951	5.1	14.3	0.0	0.9	1.8	3.6	334.7
	Costa Rica	3144	4.9	15.5	0.0	0.6	1.6	3.6	335.0
	Panama	982	10.5	33.2	0.0	0.9	2.3	5.5	352.5
	Peru	1667	5.3	26.0	0.0	0.5	1.1	2.8	653.2

### Proportion of foods meeting the 2022 PAHO regional sodium targets by categories and country

**Fig 1:**
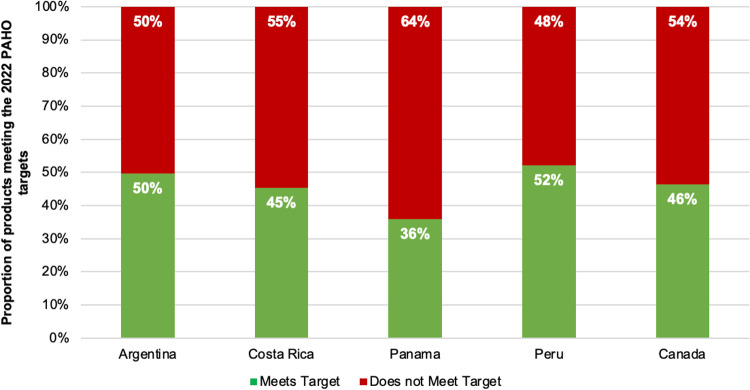
Proportion of products meeting the updated 2022 PAHO Sodium Targets – by country.

Overall, 11,037 of 23,663 (47%) analyzed foods met their respective 2022 PAHO sodium targets (mg/100g). Peru and Argentina had the highest proportion of compliance (52% and 50%, respectively), whereas Panama had the lowest (36%) (Fig 1). By food category, the highest proportion of products meeting the regional target was among *‘ready-made foods’* (77%), *‘processed meat and poultry’* (53%) and *‘cheese’* (50%). The lowest proportion of foods meeting the regional target was among *‘bread products’* (30%), *‘granola and energy bars and nut butters/spreads’* (37%) and *‘processed fish and seafood’* (41%). The largest variation between countries was observed in *‘soy products and meat alternatives’* (range: 28% to 85%) and *‘soups’* (range: 31% to 54%) (Table 3).

**Table 3:** Proportion of products meeting the 2022 PAHO Sodium Reduction Targets (mg per 100g/mL), at the regional and country level, by food category.

PAHO 2021 Major and Subcategory	Regional		Argentina		Costa Rica		Panama		Peru		Canada	
**n**	**% (n) 2022**	**n**	**% (n) 2022**	**n**	**% (n) 2022**	**n**	**% (n) 2022**	**n**	**% (n) 2022**	**n**	**% (n) 2022**
1. Bread, bread products and crisp breads	1564	30 (476)	226	27 (60)	188	36 (68)	44	20 (9)	87	55 (48)	1019	29 (291)
2. Cakes, biscuits, pastries and sweet breads	3530	43 (1526)	626	56 (351)	512	42 (217)	99	34 (34)	265	51 (134)	2028	39 (790)
3. Corn derivatives	58	47 (27)	0	NA	43	44 (19)	8	25 (2)	7	86 (6)	0	NA
4. Breakfast cereal	1089	46 (499)	87	56 (49)	195	49 (96)	48	38 (18)	142	56 (79)	617	42 (257)
5. Savoury snacks	1692	45 (754)	143	23 (33)	336	52 (174)	92	36 (33)	159	62 (99)	962	43 (415)
6. Cheese	1813	50 (909)	256	69 (176)	214	57 (121)	68	44 (30)	108	60 (65)	1167	44 (517)
7. Processed vegetables, beans, and legumes	1976	43 (852)	151	41 (62)	394	45 (178)	109	28 (30)	78	54 (42)	1244	43 (540)
8. Processed meat and poultry	1776	53 (948)	178	42 (75)	184	31 (57)	91	47 (43)	113	65 (74)	1210	58 (699)
9. Processed fish and seafood	946	41 (385)	54	54 (29)	175	42 (73)	72	36 (26)	66	33 (22)	579	41 (235)
10. Soy products and meat alternatives	274	46 (127)	77	69 (53)	50	28 (14)	5	80 (4)	26	85 (22)	116	29 (34)
11. Soups	715	48 (346)	45	53 (24)	83	40 (33)	48	40 (19)	54	15 (8)	485	54 (262)
12. Ready-made foods, convenience foods, and mixed dishes	2133	77 (1638)	144	81 (116)	116	68 (79)	24	67 (16)	65	82 (53)	1784	77 (1374)
13. Fresh or dried plain pasta and noodles	1108	43 (479)	202	16 (33)	125	57 (71)	39	41 (16)	171	41 (70)	571	51 (289)
14. Granola and energy bars and nut butters/spreads	967	37 (357)	33	67 (22)	117	50 (58)	31	35 (11)	50	68 (34)	736	32 (232)
15. Fats and oils	1044	40 (414)	86	57 (49)	144	50 (72)	48	19 (9)	74	47 (35)	692	36 (249)
16. Sauces, dips, gravy and condiments	2978	44 (1300)	203	57 (116)	341	39 (132)	153	34 (52)	223	40 (89)	2058	44 (911)
Total	23663	47 (11037)	2511	50 (1248)	3217	45 (1462)	979	35 (352)	1688	52 (880)	15268	46 (7095)

Note: n, number of foods sampled and % (n) of products meeting the 2022 PAHO Sodium Reduction Targets (items were compared against the targets set for respective sub categories). NA, not applicable.

After excluding 236 products in categories that have no available targets, 23,000 foods were included in the analysis of compliance with the PAHO SRTs for sodium levels per kcal. Overall, 10,304 of 23,000 (45%) foods met their respective sodium targets (mg/kcal). Peru had the highest proportion of compliance (52%), followed by Costa Rica (48%), whereas Panama only had 37% compliance (Table 4). By category, the highest proportion of products meeting the regional target was among *‘savoury snacks*’ (71%), *‘soups’* (67%) and *‘corn derivatives’* (67%). The lowest proportion of foods meeting the regional target was among *‘bread products’* (31%), *‘cheese’* (35%) and *‘ready-made foods’* (35%). The largest variation between countries was observed in ‘soy products and meat alternatives’ (range: 33% to 80%) and ‘fresh or dried plain pasta and noodles’ (range: 16% to 55%) (Table 4).

**Table 4:** Proportion of products meeting the 2022 PAHO Sodium Reduction Targets (mg/kcal), at the regional and country level, by food category.

PAHO 2021 Major and Subcategory	Regional		Argentina		Costa Rica		Panama		Peru		Canada	
**n**	**% (n) 2022**	**n**	**% (n) 2022**	**n**	**% (n) 2022**	**n**	**% (n) 2022**	**n**	**% (n) 2022**	**n**	**% (n) 2022**
1. Bread, bread products and crisp breads	1556	31 (487)	226	27 (62)	187	40 (74)	45	24 (11)	87	55 (48)	1011	29 (292)
2. Cakes, biscuits, pastries and sweet breads	3524	43 (1526)	625	52 (326)	511	41 (212)	99	38 (38)	265	52 (139)	2024	40 (811)
3. Corn derivatives	58	67 (39)	NA	NA	43	65 (28)	8	62 (5)	7	86 (6)	NA	NA
4. Breakfast cereal	1090	40 (441)	87	48 (42)	195	43 (84)	49	33 (16)	142	55 (78)	617	36 (221)
5. Savoury snacks	1691	71 (1205)	143	57 (82)	336	74 (248)	93	66 (61)	159	84 (134)	960	71 (680)
6. Cheese	1807	35 (639)	256	36 (92)	214	43 (93)	68	43 (29)	107	41 (44)	1162	33 (381)
7. Processed vegetables, beans, and legumes	1706	47 (795)	128	47 (60)	354	45 (161)	95	33 (31)	72	49 (35)	1057	48 (508)
8. Processed meat and poultry	1772	39 (689)	178	33 (59)	184	30 (55)	93	28 (26)	113	38 (43)	1204	42 (506)
9. Processed fish and seafood	945	48 (453)	54	52 (28)	175	57 (100)	71	41 (29)	66	56 (37)	579	45 (259)
10. Soy products and meat alternatives	272	49 (133)	77	73 (56)	49	37 (18)	5	80 (4)	26	65 (17)	115	33 (38)
11. Soups	700	67 (472)	45	40 (18)	83	48 (40)	48	44 (21)	52	67 (35)	472	76 (358)
12. Ready-made foods, convenience foods, and mixed dishes	2107	35 (735)	144	48 (69)	115	47 (54)	24	25 (6)	64	23 (15)	1760	34 (591)
13. Fresh or dried plain pasta and noodles	1110	43 (479)	201	16 (33)	128	55 (71)	39	41 (16)	171	41 (70)	571	51 (289)
14. Granola and energy bars and nut butters/spreads	967	47 (455)	33	76 (25)	117	63 (74)	32	38 (12)	49	69 (34)	736	42 (310)
15. Fats and oils	1035	43 (448)	85	52 (44)	140	41 (57)	45	29 (13)	74	39 (29)	691	44 (305)
16. Sauces, dips, gravy and condiments	2660	49 (1308)	190	53 (101)	291	40 (116)	153	27 (42)	207	48 (100)	1818	52 (948)
Total	23000	45 (10304)	2472	44 (1097)	3122	48 (1485)	967	37 (360)	1661	52 (864)	14777	44 (6497)

Note: n, number of foods sampled and % (n) of products meeting the 2022 PAHO Sodium Reduction Targets (items were compared against the targets set for respective sub categories). NA, not applicable.

### Changes in the proportion of foods meeting the old 2015 PAHO sodium targets

**Fig 2:**
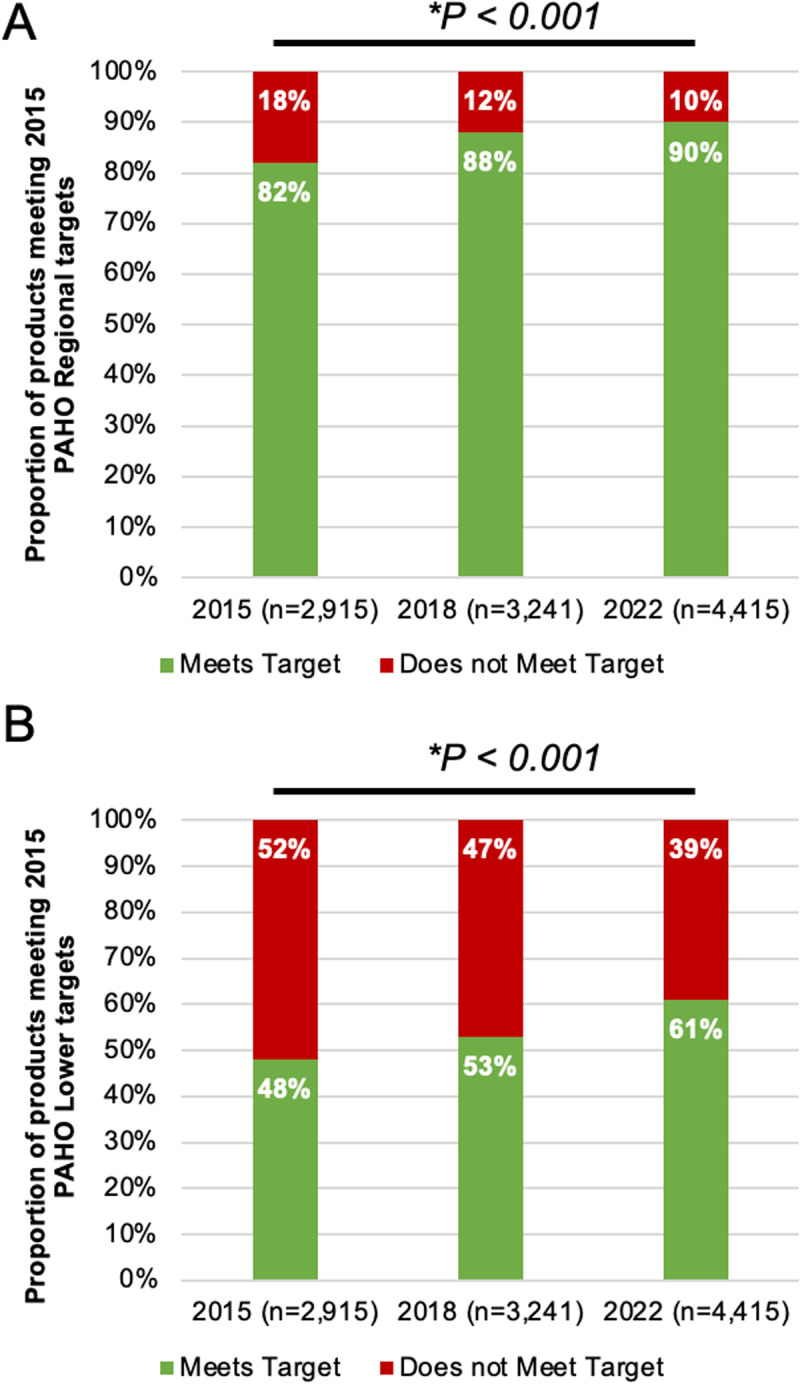
A) Proportion of products meeting the PAHO 2015 Regional Sodium Targets from 2015 to 2022 (Argentina, Costa Rica, Peru) – overall; B) Proportion of products meeting the PAHO 2015 Lower Sodium Targets from 2015 to 2022 (Argentina, Costa Rica, Peru) – overall.

**Fig 3:**
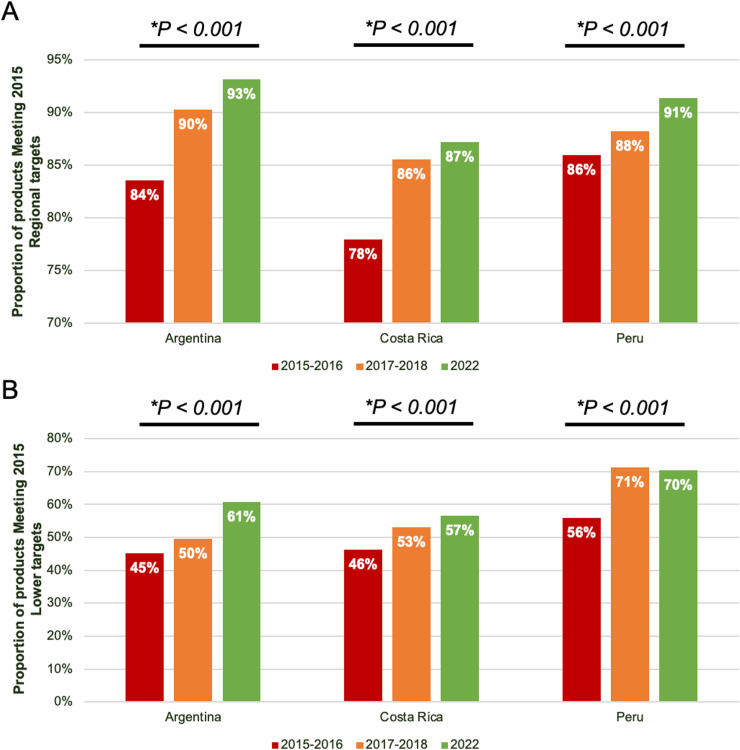
A) Proportion of products meeting the 2015 PAHO Regional Sodium Targets from 2015 to 2022 – by country (2015 – 2022); B) Proportion of products meeting the 2015 PAHO Lower Sodium Targets from 2015 to 2022 – by country.

The longitudinal analysis included a total of 10,571 foods, of which 2,915 foods were from the 2015–2016 collection, 3,241 foods were from the 2017–2018 collection, and 4,415 foods were from the 2022 collection (countries: Argentina, Costa Rica, and Peru), covering 18 PAHO sodium food categories (2015) (Fig 2). Overall, the proportion of products meeting the 2015 PAHO SRTs across the three countries increased significantly from 2015–2016 (82%) to 2022 (90%) (Fig 2A, p < 0.001). Similarly, the proportion of products meeting the 2015 PAHO lower targets was 48%, 53% and 61% for 2015–2016, 2017–2018 and 2022, respectively (Fig 2B, p < 0.001). There was also a significant increase in the proportion of products meeting the regional targets, with increases of 9%, 9% and 5% from 2015–2022 for Argentina, Costa Rica, and Peru, respectively (Fig 3A). Additionally, there was a 16%, 11% and 14% increase in the proportion of products meeting the lower targets from 2015 to 2022 for Argentina, Costa Rica, and Peru, respectively (Fig 3B). A further breakdown by food categories is presented in Supporting information S2 Table.

## Discussion

This study provides an up-to-date assessment of sodium levels in packaged foods sold in five countries in the Americas, following the publication of the Updated PAHO Regional SRTs [12]. Our results show important variation in sodium levels among key food categories, and nearly half of the assessed products complied with 2022 Updated PAHO Regional SRTs. Additionally, when assessing compliance with previous 2015 PAHO SRTs, our results indicate that the proportion of foods meeting these targets has gradually increased from 82% to 90%. However, progress has been slow, and further improvements are needed to achieve compliance with the Updated PAHO Regional SRTs. Accelerated actions are essential to achieve sodium reduction goals. These data shed light on the progress in different countries in the region towards the current sodium reduction interventions, aligned with the WHO’s SHAKE technical package for sodium reduction [16] and other initiatives aimed at achieving the 2025 global target of a 30% relative reduction in mean population sodium intake.

The results show that ‘*processed meat and poultry’* and *‘sauces, dips, gravy, and condiments’* food categories have the highest median sodium levels per 100g. This finding is consistent with a previous regional study including 14 LAC [9], which reported a similar median sodium level for *‘processed meats’* (870mg/100g). Similarly, another regional study, including 4 LAC also showed that the highest sodium level was reported in *‘bouillon cubes and powders’, ‘meat and fish seasonings’ and ‘cured and preserved meats’* [10]. In addition to the sodium levels by weight, sodium density (mg/kcal) accounts for variations in energy consumption and has been shown to have a stronger association with blood pressure than total intake [17]. Our results indicate that *‘sauces, dips, gravy, and condiments’*, *‘soups’* and *‘processed vegetables, beans, and legumes’* had the highest sodium density. These results are expected, as these food categories generally have lower energy density.

Overall, the results also revealed large variations within food categories, suggesting that lower sodium options are available and demonstrating the technical feasibility of reducing sodium levels in these categories. This is important, as it highlights the importance of combining other strategies, such as front-of-pack labels to help consumers identify lower sodium options, social marketing campaigns to encourage consumers to select lower sodium foods, marketing restrictions for products with high sodium content, and nutrition standards for foods and beverages available in schools and other settings, to disincentivize the consumption of such products. However, the high average and median sodium levels in many categories also suggested that governments need to encourage sodium reduction at the manufacturer level through food reformulation by setting mandatory SRTs for key food categories.

When examining compliance with the 2022 PAHO SRTs, almost half of the analyzed foods met their respective sodium targets (mg/100g). At the country level, compliance rate for the 2022 PAHO SRTs were similar across countries, except for Panama, which had a lower compliance rate of 36%. On examination of the longitudinal progress of Argentina, Costa Rica and Peru, there was an 8% increase in compliance when compared to the 2015 regional target (82% to 90%) and a 13% increase when compared to the 2015 lower regional target (48% to 61%). While this may indicate some progress, it remains insufficient to achieve the target of a 30% relative reduction in mean population intake of sodium by 2025. Therefore, more effective strategies, such as mandatory SRTs, are needed for better compliance with the set targets.

Furthermore, the results need careful interpretation due to the different stages at which each country is in its efforts to reduce sodium. These five countries each have national-level policies supporting sodium reduction. For example, Argentina is one of the few countries in the world to establish legal sodium maximum levels for certain food groups by passing Act 26905 in 2014, as well as educational campaigns and restaurant policies to reduce sodium intake [18]. There is also ongoing monitoring of Argentina’s sodium content in foods. Studies in 2017–2018 and 2022 showed that over 90% and 94% of surveyed food products, respectively, complied with the national sodium reduction law [19,20], similar to the current finding in compliance with the 2015 regional targets. However, this suggests that the current national limits are too lax compare with the 2022 PAHO SRTs and require further adjustment; and the necessity to include sodium source food groups that are currently not included, such as cheese and puff pastries [21].

While Costa Rica only has a voluntary sodium reduction strategy to reduce sodium levels in some key food categories, progress has been monitored continuously [22–24]. From 2013 to 2021, Costa Rica has a national plan for the reduction of sodium intake, and a private-public partnership was established in 2014 for voluntary national sodium targets. A study published by Vega-Solano et al. in 2019 showed 87% compliance with the voluntary national sodium targets [22]. The Development and Public Investment Plan 2019-2022 also contemplating the decrease in premature mortality rate due to non-communicable diseases (NCD) [24]. These policies may lead to the increased compliance rate with the 2015 sodium targets seen in 2022. In the case of Peru, there is no specific national sodium reduction strategy in place; however, Peru implemented ‘high in’ FOPL regulations in 2019 that require foods exceeding established thresholds for nutrients of concern (i.e., sugar, saturated fat, trans fat, and sodium) to display a ‘high in’ FOPL [25]. This law could indirectly motivate food industry to reformulate products in order to avoid ‘high in’ sodium labels, as has already been observed in Peru and other countries [26,27]. The food industry may have adjusted sodium content prior to this study, likely influenced by the implementation of the second phase of the FOPL policy. This could explain the relatively high compliance rate with the 2022 regional sodium targets in Peru and the significant increase in compliance rate when compared to the 2015 sodium targets.

Canada published a voluntary sodium reduction guideline for prepackaged foods in 2012 and updated the targets in 2020 [28,29]. Evaluations of this initiative have shown minimal progress over time, with only 14% of food categories meeting sodium reduction targets in 2017 [30]. In June 2022, Canada introduced its mandatory ‘high in’ FOPL regulations for saturated fat, sodium, and sugar in packaged foods [31]. With an enforcement date set in January 2026, follow-up study is necessary to examine the effect of this policy in Canada. On the other hand, Panama does not have a FOPL policy in place for products with critical excess nutrients, including sodium, which may explain, partially, the low compliance rate with regional SRTs. In December 2022, the 2022–2025 Action Plan for the Reduction of Sodium and Elimination of Trans Fats in Panama was launched after extensive consultations with key stakeholders from the governmental and non-governmental sectors, academia, civil society, and international cooperation agencies. The reformulation of processed foods, focusing on the reduction of sodium and trans fats, as well as the implementation of a FOPL are part of the strategies included in the Action Plan [32].

Overall, these interventions aiming to reduce sodium intake by encouraging food reformulations could have important public health impacts, as several simulation studies found that an important number of CVDs could have been prevented or delayed if the reformulation targets or sodium guidelines would have been met [33–36]. Worth noting that mandatory policies have shown to be more effective than voluntary initiatives. For instance, a recent evaluation in South Africa, one of the fewest countries with mandatory sodium levels for key food categories, showed a reduction in salt intake of 1.15 g/day during the first phase of the program (2015–2019), a significant reduction in population-level sodium intake, and achieved 75% compliance following the second, more stringent phase of legislation in 2019 [37,38], while other voluntary initiatives in countries like Brazil and Canada have shown only modest results in meeting sodium reduction targets [30,39,40]. Given the slow progress in meeting regional sodium reduction targets, our results highlight the importance of establishing and monitoring effective and feasible mandatory SRTs to prevent diet-related NCD deaths.

This study has both strengths and limitations that should be considered when interpreting our findings. Variations in sampling sizes among countries could potentially influence the overall study results. Therefore, a detailed examination of variations within each country at the subcategory level would be necessary, although out of the scope of this analysis. However, we do provide results of progress relative to the PAHO targets, at the major category level for each country. Additionally, while we selected packaged products from major supermarket chains, we recognize that we may not have captured all of the available packaged food products in the region, or fully represent all components of the population’s diet. Similarly, we were unable to account for consumption patterns, and further field research is needed to better understand all the sources of sodium intake of populations. Furthermore, we were unable to conduct laboratory analyses to validate the sodium concentration in all food products, as this was beyond the scope of the current analysis. Instead, we relied on the sodium values reported by food companies, as extracted from the NFt. Although Argentina and Canada have a permitted variation margin of 20% when reporting nutrient values in NFts [41,42], the accuracy could not be validated in this study. Additionally, several products per country were excluded due to the absence of sodium level declarations on food labels, as some countries within the region lack mandatory nutrient values declaration. Regional comparison at different time points was limited to only three countries, and missing data were excluded. Including other countries and matching products in future analyses will provide a more comprehensive assessment at the regional level. However, this regional analysis benefited from the utilization of common methodologies across countries (e.g., data collection, data cleaning, and analysis). Additionally, several quality assurance measures were performed including validation of food categorization and outlier checks. Furthermore, prices of products were not included in this study. To ensure the decrease in sodium content is not driven by the lower sodium products that might be higher in price (which likely will not target the majority of population), future studies should also consider including sales-weighted average sodium levels to account for the actual sales of foods sampled.

## Conclusion

Overall, this study provides an ongoing surveillance of the sodium content in package foods sold in five countries in the Americas, under key food categories of the Updated PAHO Regional SRTs. Around half of the examined foods met their respective SRTs and there has been some improvement in the compliance overtime, although the reductions have been modest at best. Further efforts are required to reach the WHO’s global sodium reduction goal by 2025, such as implementation of national mandatory SRTs, reformulation guidelines, FOPL regulations, marketing restrictions, social marketing campaigns, and nutrition standards in schools and other settings, among others.
