# Supplementary material for: Monitoring sodium content in packaged foods sold in the Americas and compliance with the updated regional sodium reduction targets
Source: PLoS One. 2025 Apr 3;20(4):e0304922. doi: 10.1371/journal.pone.0304922 (PMC11967937; doi:10.1371/journal.pone.0304922)
Supplement: Attachment — Submitted filename: Rebuttal Letter.pdf [file pone.0304922.s005.pdf]

Nutritional Sciences
UNIVERSITY OF TORONTO

WHO Collaborating Centre on Nutrition Policy for Chronic Disease Prevention

Medical Sciences Building, Room 5368
1 King's College Circle
Toronto, ON Canada M5S 1A
Tel: (416) 946-7545
E-mail: mary.labbe@utoronto.ca

Rebuttal Letter

May 21, 2024
Dear Jeanelle,

This is in response to the previous submission PONE-D-24-14316. In this new submission (PONE-D-24-19881), we did not change anything in the original submitted documents. We have now included the Human Subject Research Checklist as per request.

Please let us know if you need anything else.

Original Letter:

PONE-D-24-14316
Monitoring sodium content in packaged foods sold in the Americas and compliance with the Updated Regional Sodium Reduction Targets

Dr Mary R. L’Abbé

Dear Dr. L’Abbé,

We've checked your submission and before we can proceed, we need you to address the following issues:1. Please complete all items on the Human Participants Research Checklist that are relevant for your submission, by following this link:
http://journals.plos.org/plosone/s/file?id=dc11/PLOSOne_Human_Subjects_Research_Checklist.docx (Contact us at plosone@plos.org if you cannot access the document.) There may be overlap between the checklist items and other queries listed below; please address any duplicated queries both in your response email and on the checklist itself. Upload the completed Human Participants Research Checklist as file type “Other” when you re-submit your manuscript. This document is for internal journal use only and will not be published if your article is accepted. The requested information will help us to assess whether your submission complies with PLOS ONE’s policies and adheres to applicable reporting standards. Note that your manuscript may be rejected if you provide incomplete or inadequate responses to the checklist questions and that changing the ‘Section/Category’ of your article does not affect this requirement.We've returned your manuscript to your account. Please resolve these issues and resubmit your manuscript within 21 days. If you need more time, please email the journal office at plosone@plos.org. We are happy to grant extensions of up to one month past this due date. If we do not hear from you within 21 days, we will withdraw your manuscript.

Please log on to PLOS Editorial Manager at https://www.editorialmanager.com/pone/ to access your manuscript. You will find your manuscript in the 'Submissions Sent Back to Author' link under the New Submissions menu. Be sure to remove your previous manuscript file if you are uploading a new file in response to these requests. After you've made the changes requested above, please be sure to view and approve the revised PDF after rebuilding the PDF to complete the resubmission process.We are requesting these changes to comply with the PLOS ONE submission guidelines (https://journals.plos.org/plosone/s/submission-guidelines). Please note that we won't send your manuscript for review until you have resolved the above requests.Thank you for submitting your work to PLOS ONE and supporting our mission of Open Science.

Kind regards,

Allyza Magadan

We thank you for considering this submission and look forward to your review of our work.

Sincerely,

Dr. Mary R. L'Abbé, CM, PhD
Professor Emeritus,
Department of Nutritional Sciences,
University of Toronto
Director WHO Collaborating Centre on Nutrition Policy for Chronic Disease Prevention
